# Quantifying stability in gene list ranking across microarray derived clinical biomarkers

**DOI:** 10.1186/1755-8794-4-73

**Published:** 2011-10-14

**Authors:** Sebastian Schneckener, Nilou S Arden, Andreas Schuppert

**Affiliations:** 1 Bayer AG, Bayer Technology Services, 51368 Leverkusen, Germany; 2 Aachen Institute for Computational Engineering Sciences, RWTH, Schinkelstrasse 2, Rogowski Building, 52062 Aachen, Germany; 3 The Johns Hopkins University, Department of Applied and Computational Mathematics, Applied Physics Laboratory, Laurel, MD 20723, USA; 4 NovoCatalysis LLC, Palo Alto, CA 94306, USA

## Abstract

**Background:**

Identifying stable gene lists for diagnosis, prognosis prediction, and treatment guidance of tumors remains a major challenge in cancer research. Microarrays measuring differential gene expression are widely used and should be versatile predictors of disease and other phenotypic data. However, gene expression profile studies and predictive biomarkers are often of low power, requiring numerous samples for a sound statistic, or vary between studies. Given the inconsistency of results across similar studies, methods that identify robust biomarkers from microarray data are needed to relay true biological information. Here we present a method to demonstrate that gene list stability and predictive power depends not only on the size of studies, but also on the clinical phenotype.

**Results:**

Our method projects genomic tumor expression data to a lower dimensional space representing the main variation in the data. Some information regarding the phenotype resides in this low dimensional space, while some information resides in the residuum. We then introduce an information ratio (IR) as a metric defined by the partition between projected and residual space. Upon grouping phenotypes such as tumor tissue, histological grades, relapse, or aging, we show that higher IR values correlated with phenotypes that yield less robust biomarkers whereas lower IR values showed higher transferability across studies. Our results indicate that the IR is correlated with predictive accuracy. When tested across different published datasets, the IR can identify information-rich data characterizing clinical phenotypes and stable biomarkers.

**Conclusions:**

The IR presents a quantitative metric to estimate the information content of gene expression data with respect to particular phenotypes.

## Background

### Motivation

The challenge to identify stable tumor prognosis and predictive outcome markers remains critical in clinical cancer research. Many studies rely on microarrays to determine which genes are predominantly indicative of clinical cancer phenotypes or prognosis. However, biological and technical variations across samples and studies make it challenging to identify true, predictive clinical biomarkers [1,2]. Identification of stable gene expression signatures can facilitate the classification of clinical phenotypes and their associated physiological states. Histologic tumor grade, ER (estrogen receptor) status and predicted risk of relapse are among the currently used labels to distinguish prognosis and treatment regimes. Our motivation in this study was to determine when stable predictive biomarkers can be identified from multiple microarray studies or meta-analyses.

### Dimension Reduction

Results from microarray experiments can be arranged as an *n *by *p *matrix with *n *being the number of samples and *p *the number of measured features or probesets. *n *tends to be much smaller than *p*. Dimension reduction techniques are widely used to reduce the dimensionality of the data from *p *to a smaller value *d *[3,4]. The resulting projection represents information which classifies cells and tissues relative to physiological states and phenotypes [5].

Various methods can be used to identify large scale patterns that comprise genomic subspaces. These subspaces can then be utilized to extract biologically significant information from the genome. For example, linear projection algorithms such as SVD, PCA, ICA, or factor analysis and less commonly applied nonlinear methods such as non-negative matrix factorization (NMF) can be utilized in mapping and assessing differential behavior across large-scale genomic data [6-11]. The result is a clearer picture of the role differential gene regulation has on cellular phenotypes and the potential to identify predictive genes for disease diagnosis or prognosis. Such analyses are then critical to understanding cellular physiology, clinical phenotypes and for predicting the efficacy of drugs on diseased cells.

### Data sets and Analysis

In our analysis, published data from eight breast cancer studies, one lung cancer and one prostate cancer study were analyzed (Table 1) [12-21]. All redundant samples were removed and all expression values were mapped to corresponding gene symbols. Our analysis was restricted to genes that were present across all studies. The degree of recorded clinical information varies between studies with at most 5 phenotypic variables recorded (Table 1) per study. This allowed for a total of 87 pair-wise comparisons between studies regarding a specific phenotype. Details on data preparation and available clinical parameters are described below in the Methods section.

**Table 1:** Data sets used for this study with ArrayExpress identifiers, literature references and available meta data.

Array Express ID	Reference	Tissue	Sample size used	Clinical marker recorded	Affymetrix Platform
E-GEOD-10072	[16]	Lung	107	Tumor/control, Smoking	HG-U133A
E-GEOD- 6919	[13]	Prostate	171	Tumor/control	HG-U95Av2, B, C
E-GEOD- 6532	[18]	Breast	138	Grade, Size, Age, ER, relapse	HG-U133A, HG-U133B, HG-U133_Plus_2
E-GEOD- 7390	[15]	Breast	198	Grade, Size, ER, relapse	HG-U133A
E-GEOD-11121	[19]	Breast	200	Grade, Size, relapse	HG-U133A
E-TABM- 158	[14]	Breast	130	Grade, Size, Age, ER, relapse	HG-U133A
E-GEOD- 4922	[17]	Breast	249	Grade, Size, Age, ER	HG-U133A, HG-U133B
E-GEOD- 2990	[20]	Breast	189	Grade, Size, Age, ER, relapse	HG-U133A
E-GEOD- 5847	[12]	Breast	95	ER	HG-U133A
E-GEOD- 2034	[21]	Breast	286	ER, relapse	HG-U133A (preprocessed data downloaded)

## Results

The following section presents the results of the analysis of several publicly available microarray datasets. For each dataset the normalized expression values were projected to a lower dimensional (*d *= 4) space. Differential expression and corresponding p-values of differential expression were calculated in projected and residual space for a series of phenotypic variables. Hence, for each gene and combination of phenotype and study there are two p-values.

### Comparison of different clinical phenotypes

**Figure 1:**
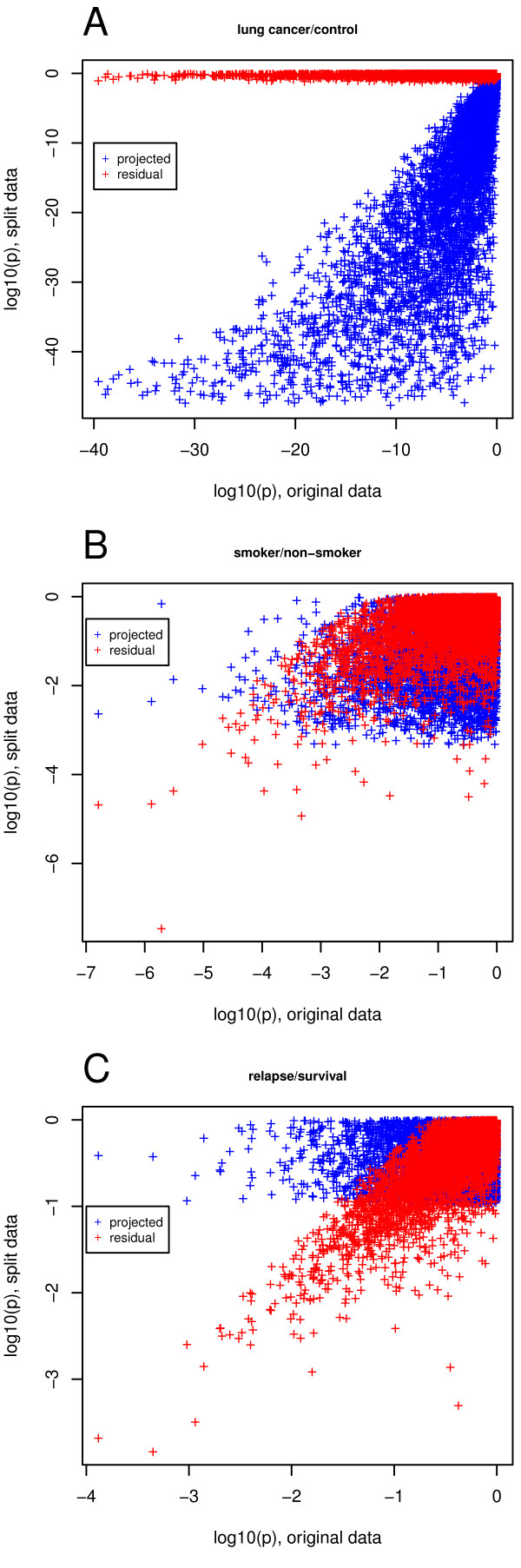
**Information partition between residual and projected space**. The data comparisons demonstrate the partitioning of information between projected data S_n _and residual data S_r _in comparison to the original data. The x-axis shows p-values of differential gene expression in the original data, while the y-axis shows p-values for projected (blue) and residual (red) data. Qualitative different types of information partitioning are demonstrated: (a) Type 1: control tissues are compared with lung cancer samples, (b) Type 2: non-smoker (no stress response) lung tissue is compared with smoker (stress response) samples, (c) Type 3: metastatic breast cancer tissue compared with non-metastatic samples.

Comparing the p-values of the projected expression value and the residual expression value can bring light to where information lies in the measured expression data. The information may reside mainly in the residual space, mainly in the projected space, or somewhere in between. A comparison of log_10 _p-value of differential expression (referred to as lp below) data from lung and breast tissue showing different clinical phenotypes was performed as shown in Figure 1. Data structures shown in Figures 1a, b, and 1c are categorized as data projection Types 1, 2, and 3 (correlated to the information ratio (IR) which is described in detail in the Methods section) respectively so that they may be easily referred to later in the text to describe the specific type of observed information distribution.

Type 1: The projection of lower dimensionality data, lp_p _(blue crosses) onto S_n _shows high significance (low lp-values) compared to the residuals lp_r _(red crosses), almost all significance from the original data (x-axis) is expressed in lp_p_, as shown by the distribution of p-values. The ratio between the lp_r _and lp_p _(information ratio) is low (Figure 1a, shows p-values of differential expression between tumor and control tissue).

Type 2: The projection lp_p _(blue crosses) onto S_n _shows similar p-values compared to the residuals lp_r _(red crosses). The information ratio is almost 0.5 (thus half of the information is stored in the residual space) (Figure 1b, showing differential expression smoker-non-smoker).

Type 3: The projection lp_p _(blue crosses) onto S_n _shows very low absolute values compared to the residuals lp_r _(red crosses). The information ratio is almost 1 (thus most of the information is stored in the residual space). (Figure 1c, shows differential expression between mammacarcinoma leading to post-surgical metastasis and no metastasis). Observe that the p-values are high compared to the other cases. Therefore, the overall information content of the expression data is low with respect to the phenotype.

The principal components are sorted in decreasing order of variance explained. The projections of differential expression onto the first principal components quantify whether the changes in the phenotype can be associated with a combination of the main data variations in the entire sample. Therefore, if in a well-controlled experiment, the sample is homogeneous (e.g. a monoclonal cell culture study using the same protocols) and only one well-defined experimental variation is performed, then all differential expressions should represent only the biological variation in the sample and should be associated with the first principal component of the PCA. This correlates with a Type 1 genome-wide differential expression pattern where the resulting distribution is dependent on the study design. In contrast, clinical studies have a high biological heterogeneity, which is not well characterized *a priori*. The type of differential expression pattern then depends on whether phenotypic changes are a result of a mixture of expression variations in the sample. Therefore, study design weighs heavily on the type of distribution observed. In our analysis, 6 breast cancer studies (E-GEOD-6532, E-GEOD-7390, E-GEOD-11121, E-TABM-158, E-GEOD-2990, E-GEOD-2034) (Table 1) showed either Type 3 (2 out of 6) or Type 2 (4 out of 6) behavior for relapse. Thus, in contrast to well-controlled laboratory experiments, data from clinical studies do not represent the expected biological/clinical variations *a priori *as they are hidden behind signals from biological heterogeneity. Therefore, a method to quantitatively translate results from lab experiments into clinical settings can be useful.

### The Information Ratio (IR)

In order to quantify the patterns we introduce the information ratio (IR). The IR describes the ratio of differential expression, which is stored in the residual space, compared to the information in both the residual and projected space. However, rather than using fold change values, p-values of differential expression are used. In order to suppress false results from genes with low overall differential expression, the IR is calculated as weighted sum of p-value ratios:

IR=∑wi log(pr,i)∕(log(pr,i)+ log(pp,i))∑wi

where *p*_*r, i *_is the p-value of the residual for gene *i*, and *p*_*p, i *_is the p-value of principle component projections for differential expression of gene *i*. The weights, *w*_*i*_, for each gene *i *guarantee that the genes with high sensitivity contribute more to IR than genes with low sensitivity. Here we use an intrinsic weight distribution so that all gene groups with similar sensitivity contribute equally to the IR.

### Calculating the Information Ratio (IR) for Different Phenotypes

**Figure 2:**
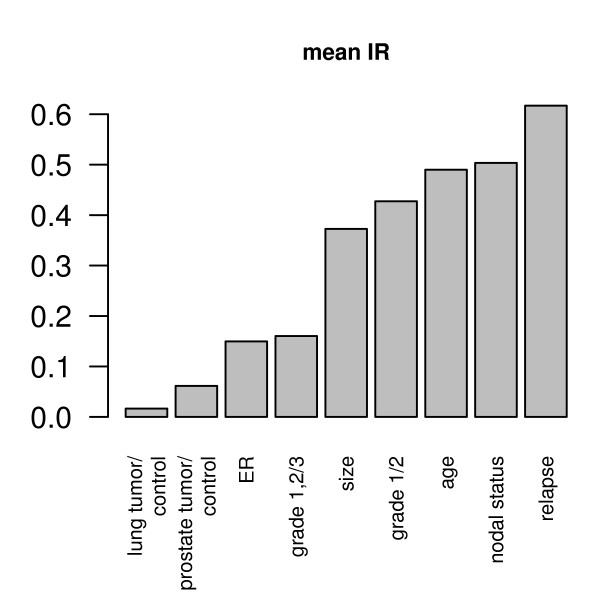
**Mean information ratios for differential phenotypes across the studies**. Low IR values are obtained for e.g. tumor vs. control lung tissue or mamma carcinoma grade 1 or 2 vs. grade 3. Higher IR values are seen in e.g. relapse vs. relapse-free.

The IR is calculated for different phenotypes and reveals a property specific to the clinical phenotype (Table 2). As seen in Figure 2, data can be categorized into high or low IR, where low IR coincides with Type 1 data projections (Figure 1a) and high IR coincides with Type 2 and Type 3 data projections (Figure 1b and 1c).

**Table 2:** Phenotypes identified by IR values where low IR values correspond with Type 1 and high IR values correspond with Types 2 and 3 data structures.

Differential Phenotypes	
**Low IR**	**High IR**

• Tumor control in lung/prostate	• Smoker/non smoker in healthy lung tissues
• Grade 1&2 versus grade 3 tumors in mammacarcinoma	• Age < 55a versus age >55a in mamma carcinoma
• ER positive versus ER negative mammacarcinoma	• Relapse of breast tumors after surgery
	• Grade 1 vs. grade 2

### Analysis of gene ranking stability in relation to the IR

**Figure 3:**
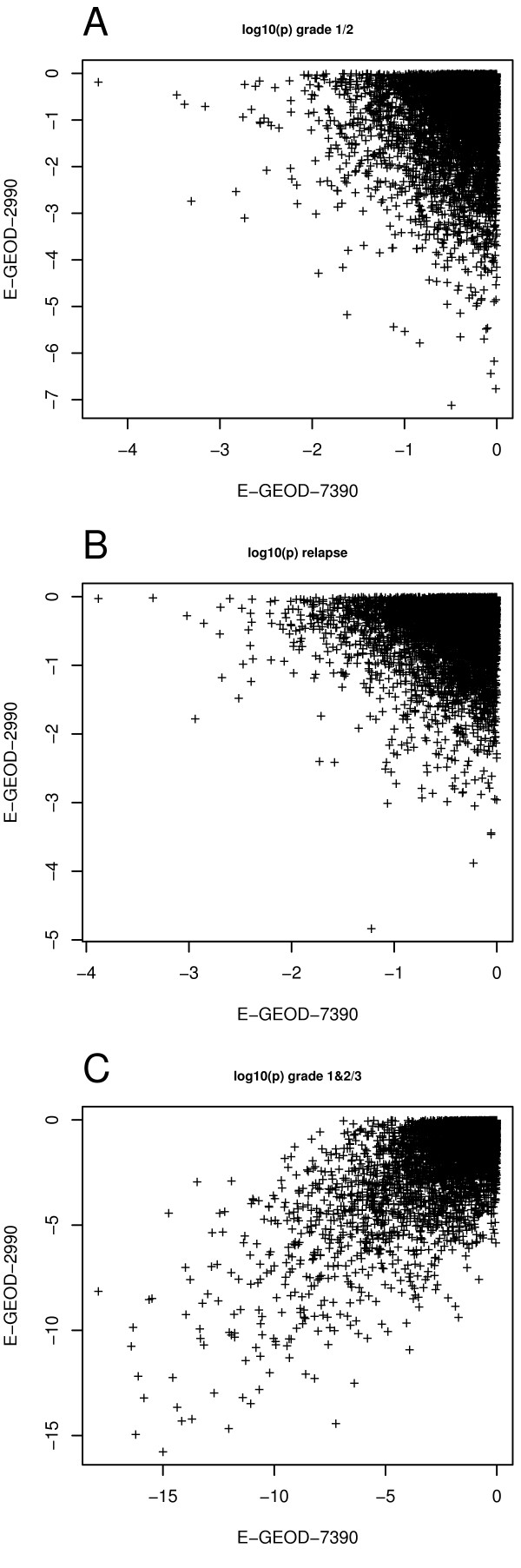
**P-values of differential gene expression compared between two studies**. Depending on the particular factor, p-values of differential gene expression may be dissimilar between studies [(a) grade 1 or 2 and (b) relapse], or similar [(c), grade 1&2 versus grade 3]. Genes that show similar differential expression in both studies are close to the diagonal.

For classification of clinical samples based on microarray data, prediction is usually performed with a gene list, a subset of all available genes. It is important to understand and gauge the stability of gene lists across different studies. To this end we used a dataset consisting of 8 breast cancer studies described in Table 1. Two of them (E-GEOD-7390 and E-GEOD-2990) are compared in detail in Figure 3. Both studies shown in Figure 3 display genome-wide distributions of differential expression, quantified by the log_10 _p-values for each gene across the pairs of tumor characteristics. As displayed for grade 1 versus grade 2 on Figure 3a, and relapse vs. non-relapse on Figure 3b, the log p-values are not related between the two studies: Genes displaying low p-values in one study are non-significant in the other study and vice versa. Thus, as shown in Figure 3a and 3b, the genome-wide distribution of information with respect to heterogeneous phenotypes is qualitatively dependent on the study. Consequently, the ranking of gene lists depends strongly on the individual study and is not easily transferable between studies.

**Figure 4:**
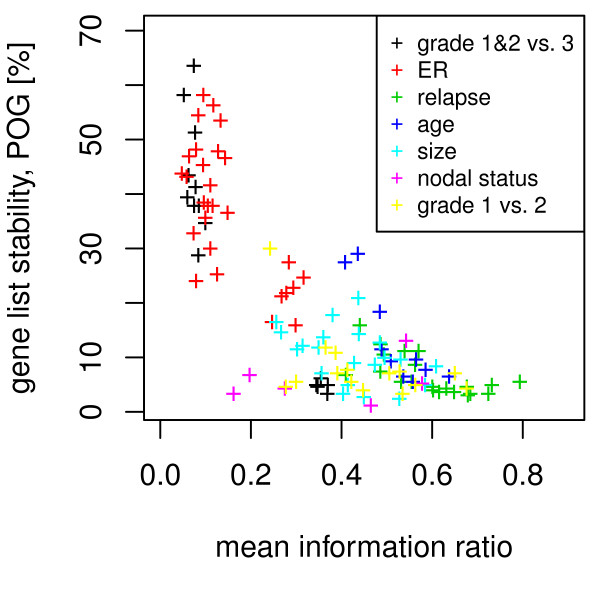
**Relationship between gene list overlap and IR**. For multiple breast cancer studies IR values of grade, size, age, ER status, and relapse are compared to the gene list overlap. Each data point represents a pair of studies with the mean IR (x-axis) and the percenatage of overlapping genes (POG) of the top 5% of p-values (y-axis).

Results shown in Figure 3c are qualitatively different from Figure 3a, b: The information carrying genes are the same in both studies. Data presented in Figures 3a and 3b, demonstrate that differentially expressed genes are not identical between studies, such that the identification of predictive biomarkers becomes almost impossible. Surprisingly we found, however, that the distributions of p-values with respect to other tumor characteristics can show a qualitatively different structure (Figure 3c). It is remarkable that Figures 3a, b and Figure 3c are based on gene expression data from the same patient cohort. The only difference is the set of physiological phenotypes (in this case, tumor grades), that are compared against their respective differential expression distributions. The differential expression pattern between grade 1 or grade 2 tumors compared to grade 3 tumors display significant similarity across both studies. This is in contrast to the distribution between grade 1 versus grade 2 tumors. Based on results shown in Figures 3 and 4, it is clear that, depending on how the phenotypic data is combined, we identify either more or less significance in the p-value comparisons across the studies. Therefore, the magnitude of agreement between gene expression studies depends less on the study design, but appears to be related to biological phenotype.

A detailed analysis of gene list stability and IR for seven factors and all 8 breast cancer studies is displayed in Figure 4. Gene list stability is quantified by the intersection between the two top-5% gene lists of a study pair. Factors associated with high or medium IR values display low degrees of gene list stability between studies and are unlikely to yield stable biomarkers. However, phenotypes associated with lower IR values show more stability and transferability between heterogeneous studies. Thus, biomarkers may be identified to discriminate between phenotypes among the low IR values.

### The Effect of Sample Size on Gene Ranking Stability

**Figure 5:**
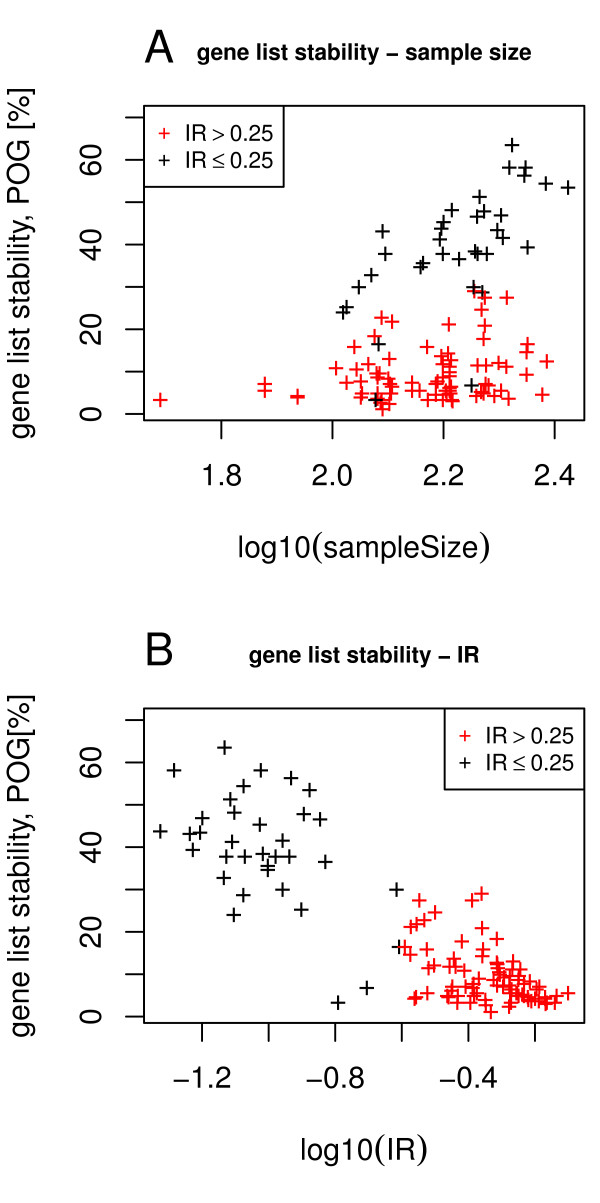
**Gene list stability**. Sample size can determine the stability of rank gene lists in most cases. The y axis is the percentage of overlapping genes (POG) in the top 5% list between two compared studies and the x axis displays the logarithmic sample size. (a) The black stars indicating IR values ≤ 0.25 and correlating with Type 1 phenotypic classifications, show linear and thus stable behavior whereas the red stars indicating IR values > 0.25 and correlating with Types 2 and 3 phenotypic classifications, show less uniform distribution and are thus unstable (overall r^2 ^= 0.15). (b) Gene list stability and the logarithm of the IR show a linear relation (with r^2 ^= 0.76).

Ein-Dor et al. estimated the stability of ranked gene lists in studies with respect to outcome of tumor therapies in terms of the size of the clinical study [2]. The overarching result was that at least 1000 patients must be included in a study in order to achieve a reliable stability. However, our study reveals phenotype-specific cases where this result may not hold true. Based on data shown in Figure 5, we demonstrate that the sample size plays an important role for Type 1 classifications, whereas for Type 2 and Type 3 classifications the sample size plays a minor role.

Our analysis, which considers gene list ranking with respect to various physiological phenotypes, shows that the impact of the sample size depends on the type of classification (Figure 5). The significance group was extracted from the top 5% of significant p-values of differential expression. Again, gene list stability was quantified by the proportion of overlapping genes in the top-5% gene list. As shown in Figure 5a for Type 1 classifications (IR ≤ 0.25, black stars) the stability increases almost linearly with the logarithm of the square root of the sample size. In contrast, this is not true for Type 2 and 3 classifications (IR > 0.25, red stars). This result seems to depend only on the type of classification and not on the phenotype. In contrast, Figure 5b shows that the stability of ranked gene lists depends linearly on the log_10 _(IR) (Pearson's r^2 ^= 0.76).

### The IR and predictor accuracy

**Figure 6:**
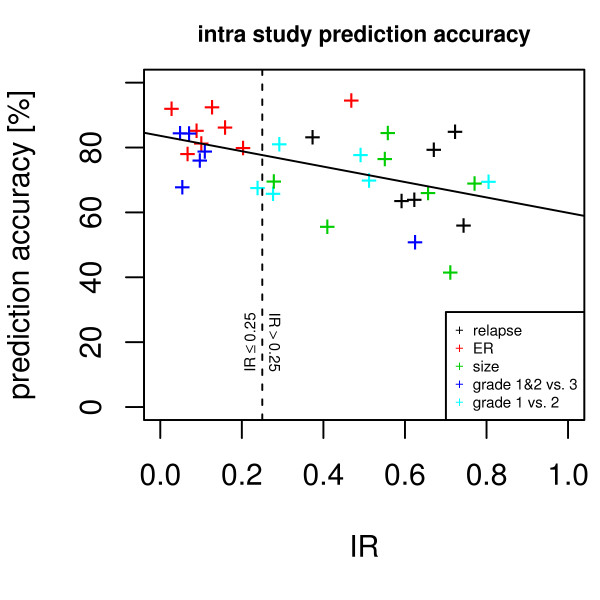
**Information ratio versus intra study prediction accuracy**. The x-axis shows the information ratio of different studies/factors. The y-axis indicates the out-of-bag prediction accuracy. The vertical dashed line delineates low and high IRs, the solid trend line indicates the decrease of accuracy with increasing IR.

The IR is a suitable indicator for gene list stability, with a high IR being indicative of a stable gene list. Non-stable gene lists are problematic for classifiers [22]. Here we evaluated the relationship between IR and the accuracy of a classifier with univariate variable selection. For ER positive vs. negative, grade 1&2 vs. grade 3, grade 1 vs. 2, tumor size large vs. small, and relapse vs. non-relapse Support Vector Machine (SVM) based classifiers were trained and accuracy on out-of-bag samples were established. The Pearson coefficient of correlation between accuracy and IR was r^2 ^= 0.25. The mean accuracy for classification tasks with IR ≤ 0.25 was 81%, while for tasks with IR > 0.25 the mean accuracy was 70%. The difference in prediction accuracy is significant with p < 0.005 (Welch two sample t-test). See Figure 6 for a detailed graphic showing the relationship between prediction accuracy and IR value.

**Figure 7:**
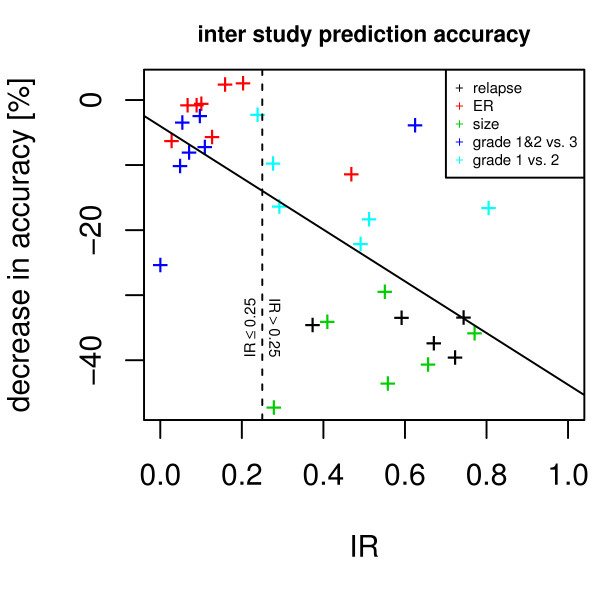
**Information ratio versus inter study prediction accuracy**. The use of biomarkers across studies decreases the prediction accuracy. The extent of accuracy loss (y-axis) depends on the IR (x-axis), as indicated by a steep descent of the solid trend line. A dashed vertical line delineates high and low IRs. Each dot represents the mean loss of accuracy for all studies when compared to the biomarker source study accuracy.

If one study is used to derive a gene list, and this gene list is used to build a classifier for another study, a decrease in accuracy can be observed. Figure 7 shows that the mean decrease for each study and factor in relation to the IR (Pearson's r^2 ^= 0.43). The mean loss of prediction accuracy is 18% for cases with IR ≤ 0.25, and 28% for cases with IR > 0.25 (p < 1e-12).

### Simulation data

A body of synthetic expression data was generated with dimensionality between 1 and 100. For this data, IR and prediction accuracy was calculated. Results demonstrate that IR and prediction accuracy depend on dimensionality which is analogous to observations in real gene expression data. However, the dependency of the IR on the specific phenotype was not apparent in the simulated data. For details on methods and results see additional file 1: Appendix 1.pdf.

## Discussion

Gene expression data sets were projected into a four-dimensional subspace and in a residual gene expression space. Depending on the phenotype the information is distributed differently between the subspace and the residual space. We introduced a p-value based information ratio, IR, to quantify this observation. When comparing cancer cells to control tissues, most information resides in the subspace (Figure 1a), however, when comparing samples from smoker to non-smokers, the information is evenly distributed between subspace and residual space (Figure 1b) and when comparing metastatic breast cancer to non-relapsing breast cancer, most information resides in the residual space (Figure 1c). The IR to quantify this observation varies between 0 and 1 with sample properties such as cancer vs. normal tissue or grade 1 and 2 vs. grade 3 result in lower IR values, whereas relapse within 5 years or patient age result in higher IR values. When using gene expression data to predict sample properties, variables related to biomarkers are selected. It has been observed that biomarkers selected from different studies may not match when sample numbers are too small. We demonstrate that the IR is indicative of biomarker stability: A low IR results in stable gene lists while a high IR results in highly unstable gene lists (Figure 4). The logarithm of the IR decreases linearly with the gene list stability (Figure 5b). Moreover, the IR is indicative of the possible prediction accuracy within a study (Figure 6). Finally, biomarker gene lists derived from low IR samples are suitable for predictions across other studies, while biomarkers from high IR samples are less reliable for predictions across studies (Figure 7).

An interpretation could be that in Type 1 classifications, where IR values are low, the genome-wide differential expression associated with the shift in the phenotype, can be expressed by a combination of a few independent leading variations in the differential gene expression pattern. These variations may be represented by biological heterogeneity and the disease-related pattern in the sample. Hence, the true dimensionality of the genome-wide differential expression pattern becomes very low, such that variation in sample size within the range of standard clinical studies will have a significant impact on the stability. In contrast, the genome-wide differential expression shift of Type 2 and 3 classifications (high IR values) cannot be reduced to the leading biological heterogeneities and hence retain high dimensionality. As the impact of sample size variation may depend on the dimensionality of the differential expression pattern, Type 2 and 3 classifications will benefit significantly less from increased sample sizes, which can be seen in typical clinical studies. Moreover, the qualitative heterogeneity of the genome-wide information distribution across different studies for high IR phenotypes indicate that biomarkers which are identified using ranked gene lists, will most likely not be predictive through statistical approaches alone. The information ratio can serve as a method to better understand the stable phenotypic variations within a study, especially since studies performed by various groups are often unable to identify stable gene lists despite the similar disease types or tissues under investigation [2,23-25]. Experiments with synthetic expression data confirm that low dimensional data yields low IR values and good prediction accuracy while high dimensional data yields high IR and poor prediction accuracy.

## Conclusion

In summary, the IR provides a metric for the capability of gene expression data to support clinical decisions. It has been observed elsewhere [22] that the predictivity of expression data depends more on the phenotype to be predicted than on the particular algorithm used. To our knowledge, the IR is the first approach to quantify this property of clinical phenotypes and it allows researchers and clinicians to clearly delineate phenotypes for which identification from gene expression data needs more sophisticated analytical methods than those which are currently widely used. Based on our study, in order to identify stable biomarkers for clinical tumor characterization, the IR should be carefully assessed. Stable predictive models across studies can only be expected if the phenotype to be predicted shows a low IR (Type 1 classification), whereas for other phenotypes the biomarker stability may be insufficient. Unfortunately, highly desirable predictive gene lists, such as those which can elucidate the prognosis of individual relapses, belong to the classification with high IR values. Thus, future progress in biomarker identification will require new approaches in both analytical methods and in clinical study design that yield more stable predictive gene lists for the high-IR classifications.

## Methods

### Analysis and Data sets

Eight breast cancer, one lung cancer, and one prostate gene expression data sets along with clinical information were downloaded from the EBI ArrayExpress website [26]. See Table 1 for details. All CEL files were uniformly processed using the MAS5 algorithm [27] as implemented in the R package simpleaffy [28]. The expression data was transformed to log_2 _values.

The sets of samples from different sources did partly overlap. In order to remove redundant measurements, the correlation of all samples with all other samples was calculated and from pairs of samples with R^2 ^≥ 0.99 one sample was omitted from this analysis. This occurred between the breast cancer studies E-GEOD-4922, E-GEOD-2990, and E-GEOD-3494. Then, to avoid a bias due to erroneous chips, samples with extreme mean expression rates (> 5σ) (one sample from E-GEOD-4922) have been omitted.

All probe set identifiers were mapped to Entrez gene symbols. In case several probe sets share the same gene symbol, the probe set with the largest mean expression over all samples was used as representative for that symbol. Across all studies, 6384 symbols were shared and only those were used for further analysis. It should be noted that probe sets representing the ER gene (ESR1 and ESR2) were included. The associated clinical information was transformed to a binary value: Grade (grade 1 or 2 vs. grade 3, resp. grade 1 vs. grade 2), tumor size (>25 mm vs. <20 mm), ER status (positive versus negative as reported), and outcome (relapse or distant metastasis free survival over five years vs. metastasis) (Table 1).

### Spectral decomposition of matrix, PCA

For each data set, the correlation matrix {C_ij_} with C_ij _being the pairwise correlation between the logarithmic expression of gene i and gene j, i, j = 1...N, was calculated. Next, a Principal Component Analysis (PCA), as implemented in MATLAB, was used to decompose {Cij} into its eigenvectors and eigenvalues, where the first eigenvectors represent the dominant, coherent variations in the data set. We denote the space, spanned by the first n eigenvectors, as S(n). Each eigenvector *k *represents a metagene whose expression X_*k, l *_in each tissue *l *is given by the weighted sum of the contribution of all genes *j *to the eigenvector:

$$X_{k,l}=\underset{j=1:N}{\sum }G_{j,k}x_{j,l}$$

This representation, using only the G_ik_-values, does not explicitly contain the data from the respective data source. However, since PCA represents the dominant variations within the respective data sets, normally the vectors G_l_, quantifying the contribution of all genes to eigenvector l, depend on the composition of the data sample.

Although the individual vectors G_1_,...,G_n _depend on the composition of the samples, the subspace S_n_, spanned by the set of all the first *n *vectors, depends significantly less on the sample composition. An appropriate value of *n *may depend on the variability studies, in the studies analyzed here *n *= 4 was used leading to sufficient results. Higher *n *did not lead to more significant differential expressions of the projections p_p, i _with respect to Type 1 classifications. This indicates that the subspace S_n _is related to biological features. Changes in sample composition merely result in a rotation of the "coordinate system" spanning S_n_, which can be represented not only by the vectors {G_1_,..., G_n_}, but also by all orthogonal vector systems which can be generated by the rotation of {G_1_,..., G_n_}.

### Split of gene expression value into original and residual values

The expression values of each gene i in each tissue k can be split into two components: a component x_p, _which is part of S_n _and a residual component, x_r_, which is part of S_r_, the subspace is then orthogonal to S_n_:

$$x_{i,k}=x_{p;i,k}+x_{r;i,k}$$

The decomposition is performed by the projection of x_i, k _onto S_n _using the solution r_i _of the following linear equation system for each gene i:

$$\left[\underset{-}{X},...,\underset{-}{X}\right]{\underset{-}{r}}_{i}={\underset{-}{x}}_{i}-<{\underset{-}{x}}_{i}>$$

Then it holds:

$${\underset{-}{x}}_{p,i}=\left[{\underset{-}{X}}_{1},...,{\underset{-}{X}}_{n}\right]{\underline{r}}_{i}$$

$${\underset{-}{x}}_{r,i}={\underset{-}{x}}_{i}-{\underset{-}{x}}_{p,i}$$

The decomposition splits each expression value for each gene in each sample into 2 components. This apparent doubling of complexity yields additional insights into the information contained in the genomic data. Then we calculate the information content of the original expression values for each gene x_i _and for both split components x_p, i _and x_r, i _with respect to different physiological or clinical phenotypes. For example, (i) we set mamma carcinoma of grade 1 and grade 2 to be class 1 and tumors of grade 3 in class 2. Next, (ii) we use the p-values of a two-sided t-test (or parameter-free Wilcoxon test) to quantify the differential expression of each gene between tissues of class 1 and class 2. Finally, (iii) we get the genomic set lp = log_10 _p, which are the logarithms of these p-values. The significance values for the projections x_p _and x_r _are then denoted as lp_p _and lp_r_, respectively.

### Weight distribution, w

**Figure 8:**
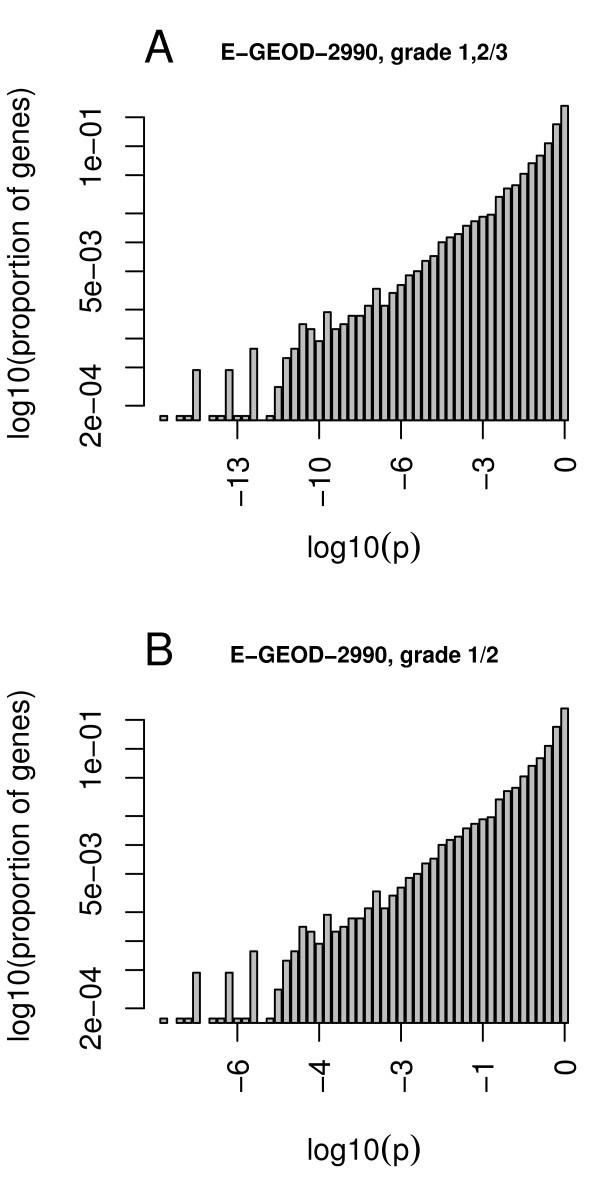
**Exponential distribution of p-values of differential expression**. The y-axis is the logarithm of the ratio of genes in the same bin of p-values with respect to all genes.

To calculate the intrinsic weight distribution, *w*, we observed that the distribution of the genomic log_10 _p-values with respect to almost all physiological factors satisfy an exponential distribution (Figures 8a, b). Figure 8 shows the histogram of the log_10 _p-values of differential gene expression for all genes, exemplified by two different endpoints. Figure 8 indicates an exponential distribution of significance over all genes. All p-values were collected and distributed over 50 equidistant bins. For each bin *j*, we calculate the ratio r_j _as the number of genes in the bin to the total number of genes throughout all bins:

$$r_{j}=\frac{events_{j}}{events_{total}}$$

Based on the observation of an exponential distribution, we use a log-linear regression model to quantify the weights:

$$w_{i}=e^{\lambda (|\log_{10}(p_{i})|)}$$

where λ is chosen such that *w *approximates the density of the respective genome-wide log(p) distribution as depicted in Figure 8.

### Information Ratio, IR

The information ratio was calculated based on lp_p _and lp_r_. Since this depends on the choice of *n*, *n *was evaluated in a range of 1 to 10. The IR decreases with increasing *n *and stabilizes at *n *= 4. This value was selected after visual inspection (see additional file 2: Appendix 2.pdf). The IR is calculated as

IR=∑wi log(pr,i)∕(log(pr,i)+ log(pp,i))∑wi

### Gene list stability

Several metrics for comparing the order of gene lists between studies are available [29], here we use the percentage of overlapping genes (POG) [30] in the top 5% of a ordered gene list. Differentially expressed genes are ordered by the p-value of a Welch's t-test statistic [31].

### Predictor accuracy

The correlation between the IR and the potential accuracy of a predictor was evaluated. For this we used SVM as implemented in the libSVM library [32] and utilities from the R packages caret [33] and e1071 [34]. We used the SVM as a classification machine with a radial basis kernel. For a given study and factor, a SVM was trained with nested 10 times 10 cross validation. The inner cross validation was used to estimate optimal gamma and cost parameters, the outer cross validation was used to select the variables. From all genes, the top 5% differentially expressed genes were used as variables. The accuracy was estimated on test-sets which were used for neither variable selection nor parameter optimization. For a given study and factor combination, the mean accuracy over the outer cross validation was established and compared to the IR (see Figure 6). A correlation between IR and mean accuracy was calculated using Pearson's correlation.

### Inter study gene list predictor accuracy

A loss in prediction accuracy can be expected when a gene list derived from one study is used for classification in another study. From the first study and factor, the top 5% differentially expressed genes were extracted. This gene list was then used to train an SVM for each study with default parameters. The out of bag prediction accuracy was established. In turn, each study was used to derive a gene list, and this list was evaluated with all the other studies. The derived accuracy for the first study was better than the mean accuracies for other studies. Figure 7 presents the decrease in mean accuracy by applying the gene lists to separate studies.

### Overview Methods

See additional file 3: Appendix 3.pdf for a graphical depiction of the analysis workflow.

## Authors' contributions

AS, NSA, and SS performed research and analysis for the paper. SS and AS conceived and designed the study. NSA wrote the paper with contributions from all authors. All authors have read and approved the final manuscript.

## Pre-publication history

The pre-publication history for this paper can be accessed here:

http://www.biomedcentral.com/1755-8794/4/73/prepub

## Supplementary Material

Additional file 1 **Simulated data**. Simulated expression data and estimation of IR and predictor accuracy for different dimensionalities of the data. Click here for file

Additional file 2 **Figure S1**. Plot of subspace dimensionality against IR. Click here for file

Additional file 3 **Workflow**. Three slides with illustrations of the used workflow to calculate the IR and predictor accuracies. Click here for file
