# Supplementary material for: Quantifying stability in gene list ranking across microarray derived clinical biomarkers
Source: BMC Med Genomics. 2011 Oct 14;4:73. doi: 10.1186/1755-8794-4-73 (PMC3206838; doi:10.1186/1755-8794-4-73)
Supplement: Additional file 1 — Simulated data. Simulated expression data and estimation of IR and predictor accuracy for different dimensionalities of the data. [file 1755-8794-4-73-S1.PDF]

Appendix 1 – Simulated data

Summary

We simulated expression data with a simple model in which we were able to vary the dimensionality of the data, but were limited from studying the biological differences across clinical factors. Despite this limitation, the resulting features of dimensionality, IR, and prediction accuracy show consistency when compared with data derived from real biological samples.

Materials and methods

Data simulation

We present a virtual set of biological samples that are characterized by *n* biological states *s*. Each state is randomly assigned from a normal distribution with mean 0 and standard deviation 1. Each sample generates expression values for 1000 ‘genes’. The gene expression is a linear combination of the states *s* of a cell and a randomly generated ‘regulation’ vector *r*, i.e. the influence of the ‘biological state’ on the expression, plus some noise. Values of *r* are randomly assigned from a normal distribution with mean 1 and a standard deviation of 3. This means that the gene expression depends on all biological states simultaneously, however, for each gene the biological states have a varied degree of contribution.

A response vector for the samples is derived from the first biological state and classes are assigned as the biological state being above or below the mean of the state for all samples.

See Figure 1 for a heatmap of gene expression with *n*=5 biological state variables, 100 samples (x-axis) and 1000 genes (y-axis). The response of interest is indicated as red/green colored bar.

Virtual data sets are generated ten times each for different *n* between 1 and 100, with *n* being the dimensionality of the data.

Predicting the response variable

The simulated data is used for training and testing a SVM as described in the main manuscript. For each data set, typical prediction accuracy is estimated from out-of-bag samples. All reported values are the mean values derived from ten virtual trials.

Calculating the IR

The IR is calculated for each data set as described in the main manuscript. Again, reported values are mean IRs over ten replicas of simulated data per dimensionality *n*.

Results

Figure 2 shows the extent to which the IR (Figure 2A) and prediction accuracy (Figure 2B) depend on the dimensionality *n* of the data set, and the correlation between IR and accuracy (Figure 2C).

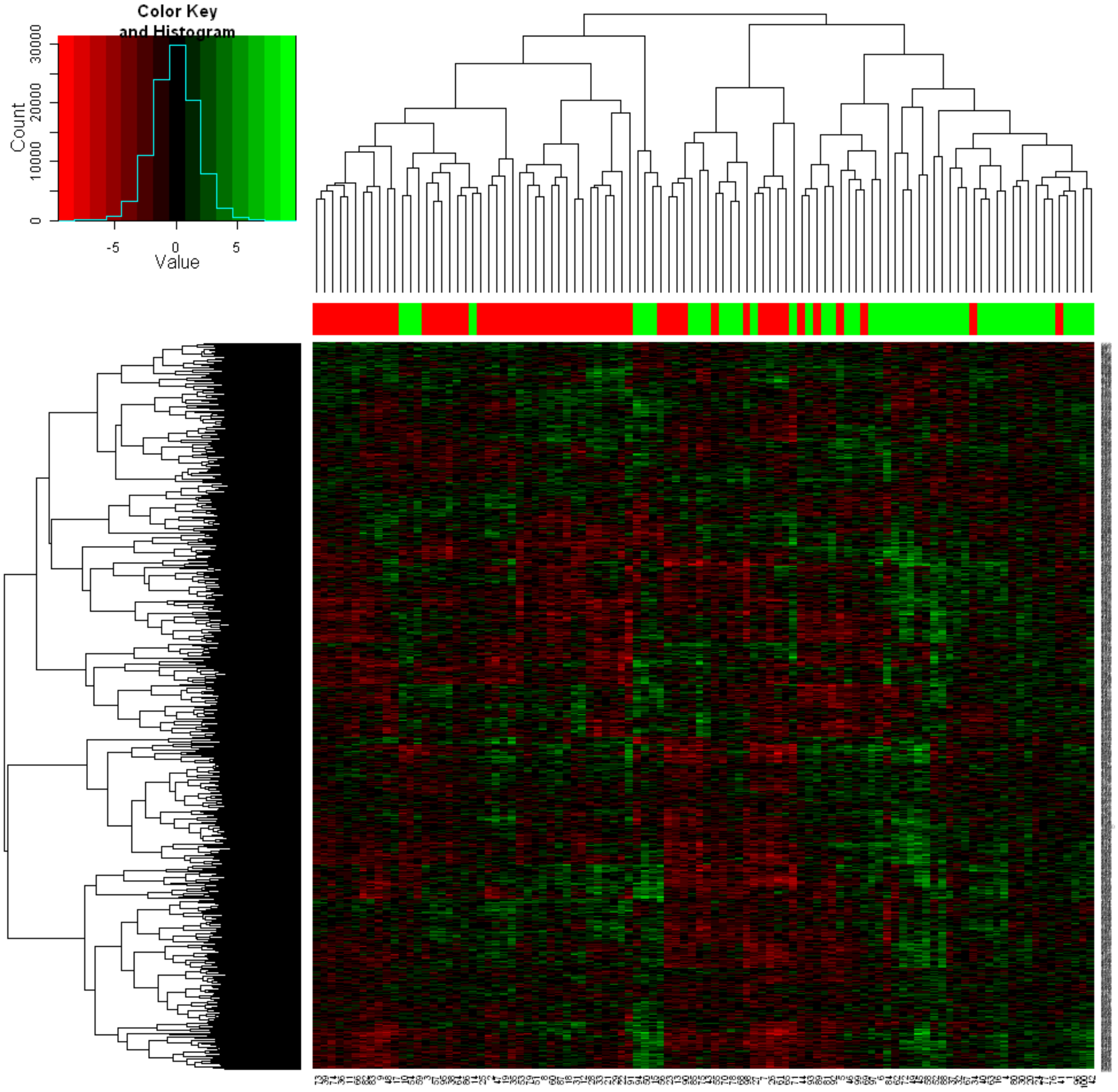

Figure 1: Heat map of simulated gene expression with 100 samples (x axis) and 1000 genes (y axis). Simulation with 5 independent biological states (dimensionality *n*=5). The red/green bar on top indicates classes (derived from a first state vector) used for prediction.

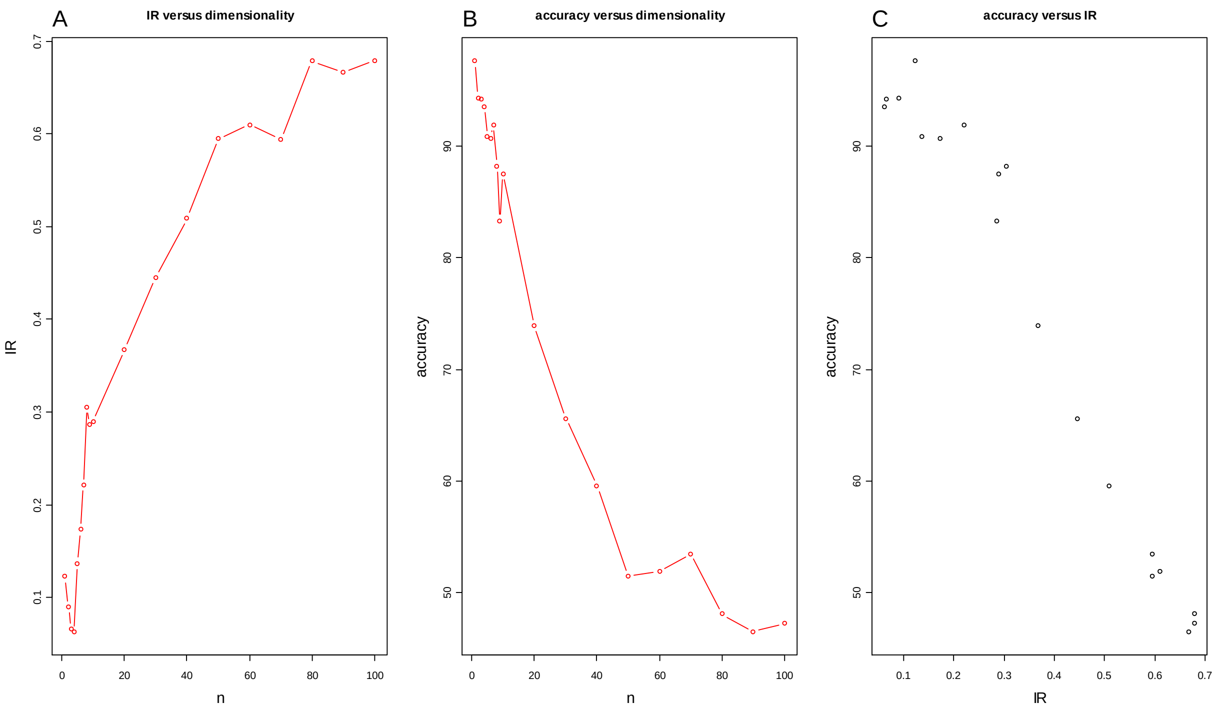

Figure 2: Properties of synthesized data with varying dimensionality. The IR depends on the dimensionality n (2A), as does the prediction accuracy (2B). (2C): Similar to biological data, the accuracy is high if the IR is low (IR<0.25). High IR values indicate that predictions accuracy is at the no information ratio level (50%).

Discussion

Controlling the dimensionality of expression data was facilitated by our data generation technique. With increasing dimensionality *n*, the response is increasingly diluted as shown by the decreasing accuracy. Likewise, we observe a correlation between the dimensionality of the simulated data set and the IR. This simulation captures the main characteristics that are observed in experimental data. However, the simulated biological states are identical to each other and thus do not distinguish between different clinical response factors (such as grade 1 vs 2 or grade 1 & 2 vs 3).
