# Supplementary material for: Quantifying stability in gene list ranking across microarray derived clinical biomarkers
Source: BMC Med Genomics. 2011 Oct 14;4:73. doi: 10.1186/1755-8794-4-73 (PMC3206838; doi:10.1186/1755-8794-4-73)
Supplement: Additional file 2 — Figure S1. Plot of subspace dimensionality against IR. [file 1755-8794-4-73-S2.PDF]

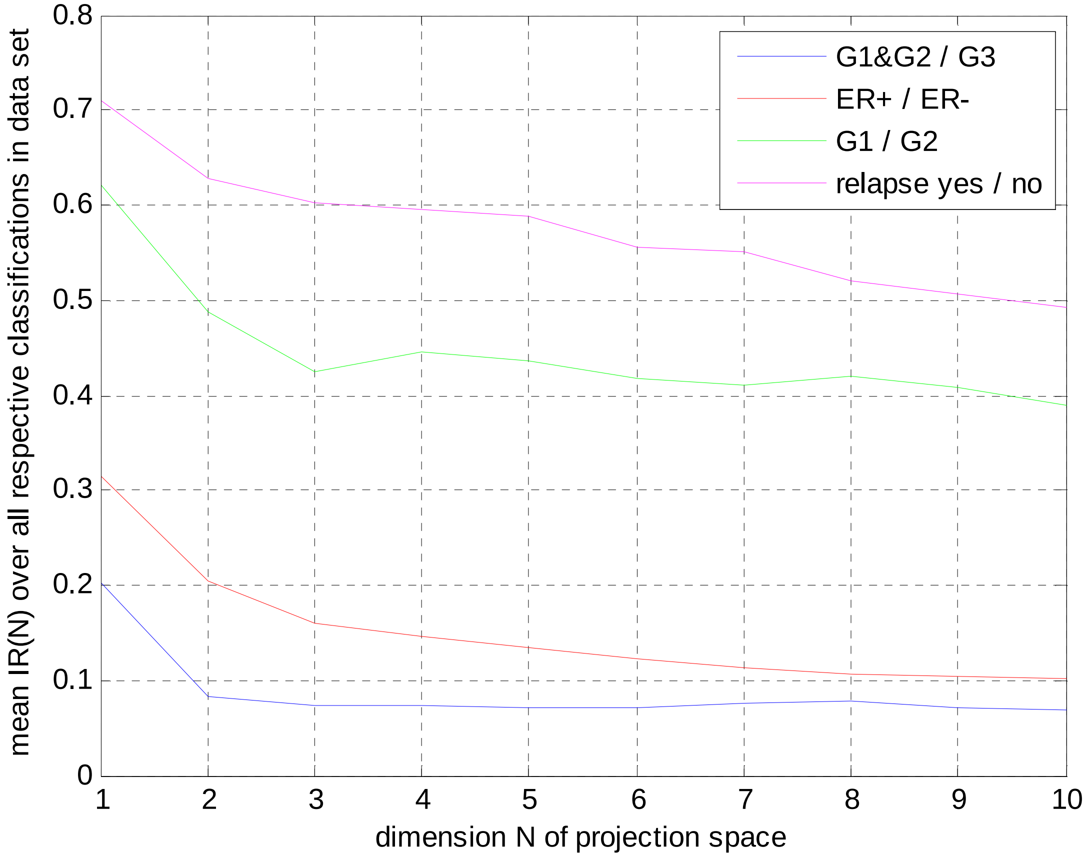

Figure S1: Plot of *n* (number of dimensions of subspace, x-axis) against the mean IR for a particular factor. The IR value stabilizes with increasing number of dimensions and after visual inspection *n*=4 is chosen for all analyses.
