# Supplementary figures and images for: Quantifying stability in gene list ranking across microarray derived clinical biomarkers
Source: BMC Med Genomics. 2011 Oct 14;4:73. doi: 10.1186/1755-8794-4-73 (PMC3206838; doi:10.1186/1755-8794-4-73)

# Workflow IR calculation

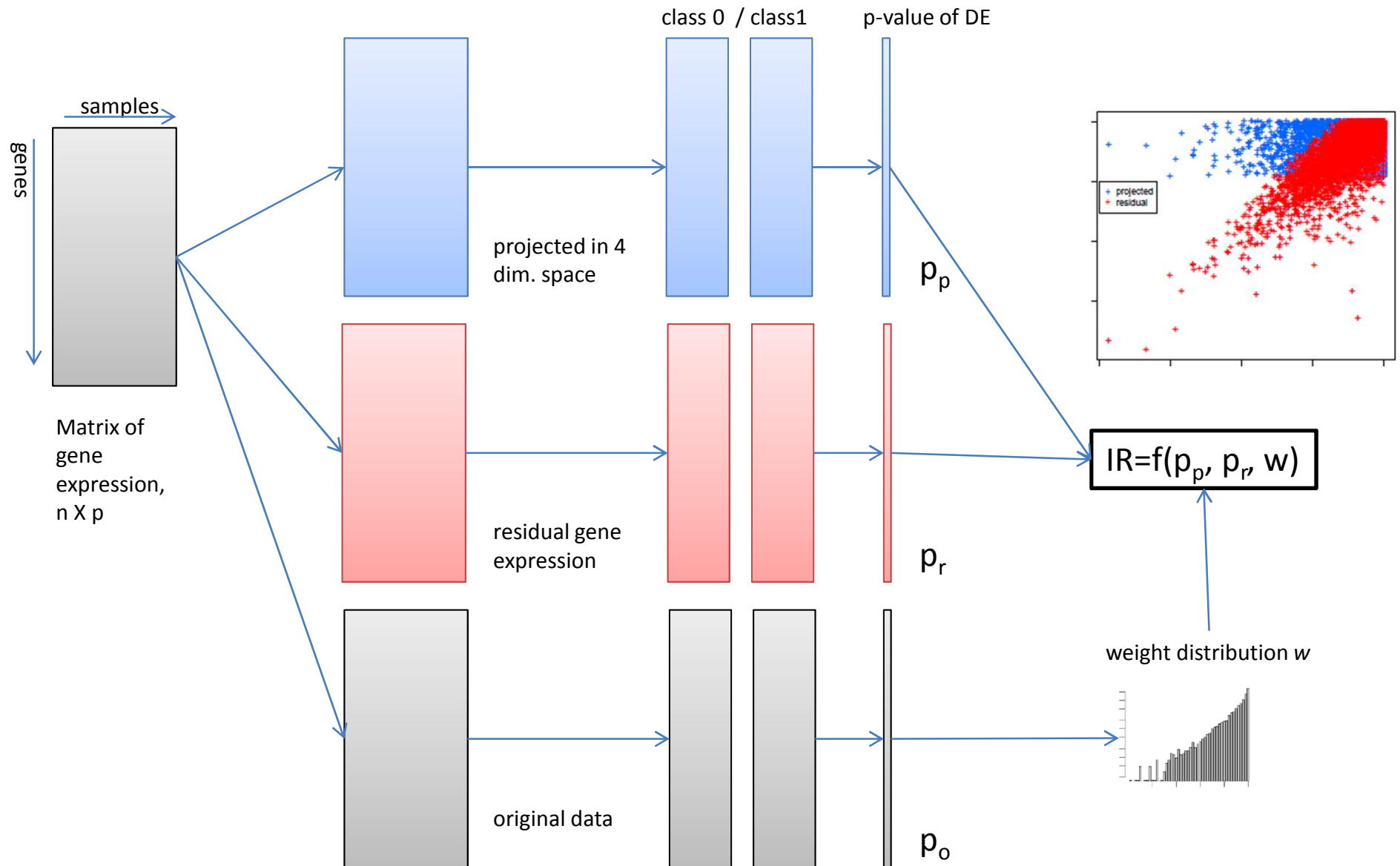

# SVM classifier – intra study

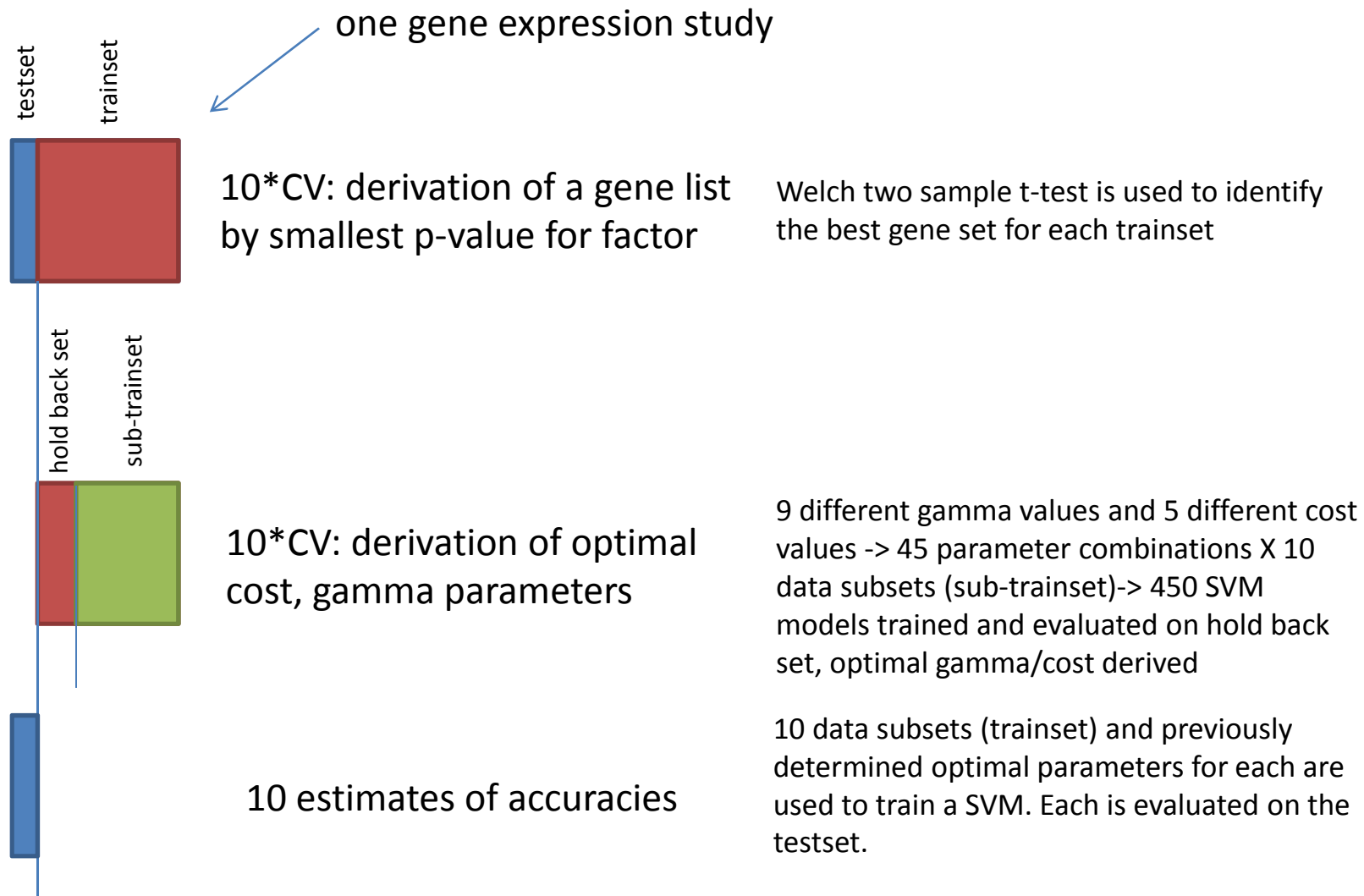

# SVM classifier – inter study

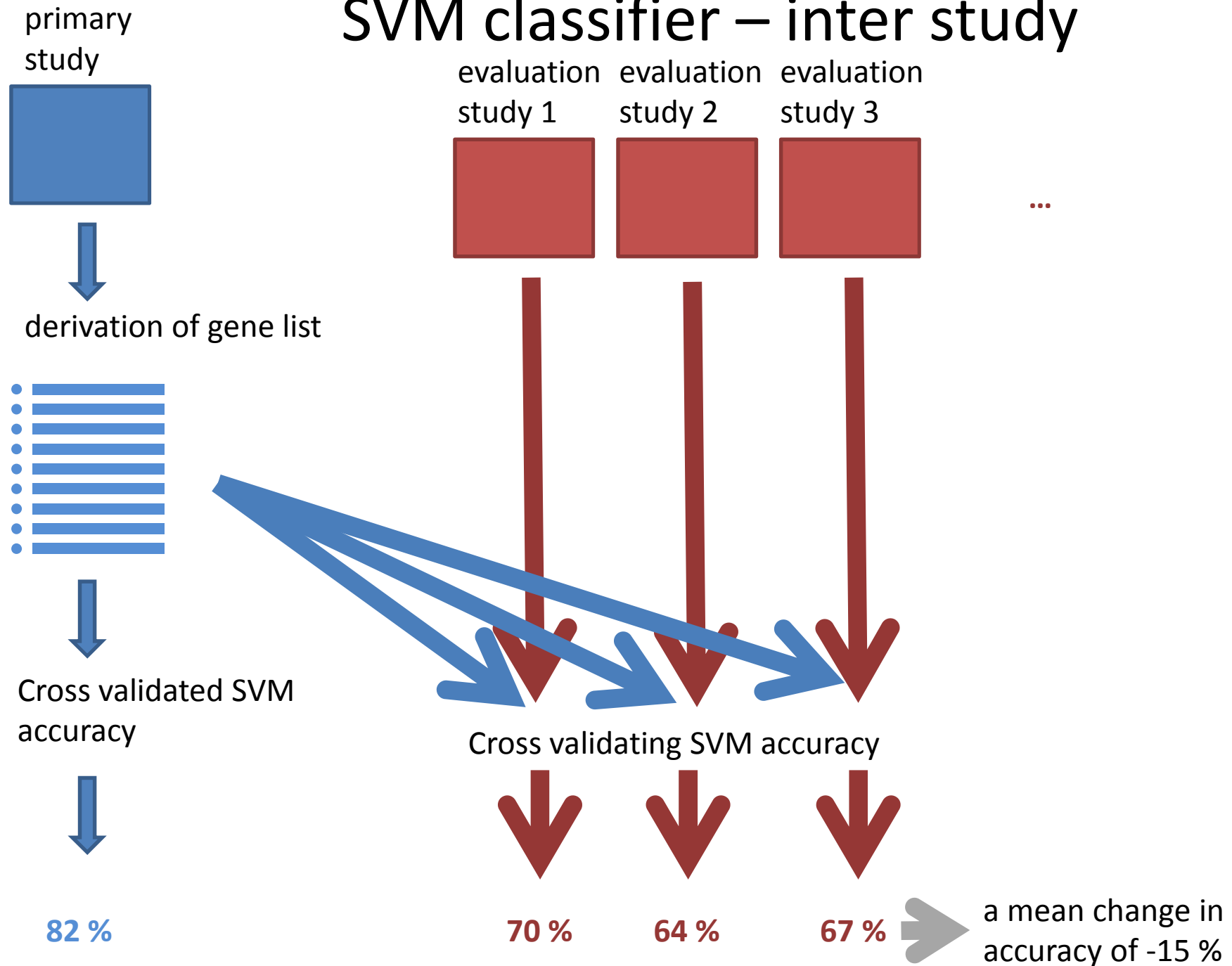

Supplement: Additional file 3 — Workflow. Three slides with illustrations of the used workflow to calculate the IR and predictor accuracies. [file 1755-8794-4-73-S3.PDF]
